# Control of the temporal development of Alzheimer’s disease pathology by the MR1/MAIT cell axis

**DOI:** 10.1186/s12974-023-02761-6

**Published:** 2023-03-21

**Authors:** Season K. Wyatt-Johnson, Holly N. Kersey, Juan F. Codocedo, Kathy L. Newell, Gary E. Landreth, Bruce T. Lamb, Adrian L. Oblak, Randy R. Brutkiewicz

**Affiliations:** 1 Department of Microbiology and Immunology, Indiana University School of Medicine, Indianapolis, IN 46202 USA; 2 Stark Neurosciences Research Institute, Indiana University School of Medicine, Indianapolis, IN 46202 USA; 3 Department of Pathology and Laboratory Medicine, Indiana University School of Medicine, Indianapolis, IN 46202 USA; 4 Department of Neurology, Indiana University School of Medicine, Indianapolis, IN 46202 USA; 5 Department of Anatomy, Cell Biology and Physiology, Indiana University School of Medicine, Indianapolis, IN 46202 USA; 6 Department of Medical and Molecular Genetics, Indiana University School of Medicine, Indianapolis, IN 46202 USA; 7 Department of Radiology and Imaging Sciences, Indiana University School of Medicine, Indianapolis, IN 46202 USA

**Keywords:** Amyloid, Innate immunity, T cell activation, Mouse models, Alzheimer’s disease

## Abstract

**Background:**

Neuroinflammation is an important feature of Alzheimer’s disease (AD). Understanding which aspects of the immune system are important in AD may lead to new therapeutic approaches. We study the major histocompatibility complex class I-related immune molecule, MR1, which is recognized by an innate-like T cell population called mucosal-associated invariant T (MAIT) cells.

**Methods:**

Having found that *MR1* gene expression is elevated in the brain tissue of AD patients by mining the Agora database, we sought to examine the role of the MR1/MAIT cell axis in AD pathology. Brain tissue from AD patients and the 5XFAD mouse model of AD were used to analyze MR1 expression through qPCR, immunofluorescence, and flow cytometry. Furthermore, mice deficient in MR1 and MAIT cells were crossed with the 5XFAD mice to produce a model to study how the loss of this innate immune axis alters AD progression. Moreover, 5XFAD mice were also used to study brain-resident MAIT cells over time.

**Results:**

In tissue samples from AD patients and 5XFAD mice, MR1 expression was substantially elevated in the microglia surrounding plaques vs. those that are further away (human AD: *P* < 0.05; 5XFAD: *P* < 0.001). In 5XFAD mice lacking the MR1/MAIT cell axis, the development of amyloid-beta plaque pathology occurred at a significantly slower rate than in those mice with MR1 and MAIT cells. Furthermore, in brain tissue from 5XFAD mice, there was a temporal increase in MAIT cell numbers (*P* < 0.01) and their activation state, the latter determined by detecting an upregulation of both CD69 (*P* < 0.05) and the interleukin-2 receptor alpha chain (*P* < 0.05) via flow cytometry.

**Conclusions:**

Together, these data reveal a previously unknown role for the MR1/MAIT cell innate immune axis in AD pathology and its potential utility as a novel therapeutic target.

**Supplementary Information:**

The online version contains supplementary material available at 10.1186/s12974-023-02761-6.

## Background

Alzheimer’s disease (AD) is the most common form of dementia in older populations [1]. However, there is still much that remains unknown about the etiology of the disease. Although AD pathology includes the build-up of amyloid-beta (Aβ), neuroinflammation, degenerating neurons, and gliosis, current treatments have not resulted in a cure for the underlying neuropathology [2, 3]. This has led to a more diverse path of exploration to fully understand all of the elements of AD. A newer focus has been on understanding what cell types are contributing to the development of neuroinflammation. Microglia have the ability to induce neuroinflammation, but other cell types, including cells from the peripheral immune system, also contribute to this inflammation. Extending from their pro-inflammatory activity, immune cells likely impact AD pathology [3]. It has previously been shown that the depletion of T cells, B cells, and natural killer (NK) cells contributes to an increase in Aβ deposition [4–6]. Adoptive transfer of T helper 1 (Th1) cells into AD model mice results in a decrease in the amount of Aβ [6]. These studies implicate a role for the adaptive immune system in AD. However, T cell activation relies on the recognition of peptide antigens in the context of major histocompatibility complex (MHC) molecules [7]. Notably, the loss of MHC class II molecules has been shown to increase Aβ plaques [3, 6]. Furthermore, aging enhances the expression of MHC class I molecules in the brain; this leads to decreased cognitive function [8]. Although these two classical peptide antigen-presenting molecules are known to be affected in AD and aging, other less well-known antigen-presenting molecules have yet to be studied in the context of AD.

The MHC class I-related molecule MR1 [9], presents microbial vitamin B-derived metabolites to an innate T cell subpopulation called mucosal-associated invariant T (MAIT) cells [10, 11]. The MR1/MAIT cell axis has been associated with a number of inflammatory disorders, including those in the CNS [11, 12]. Of note, in multiple sclerosis (MS), the number of MAIT cells is elevated, with MAIT cells producing a higher level of pro-inflammatory cytokines, concomitant with an increase in MR1 expression [10, 13]. MR1 is present on a variety of different cell types in numerous locations throughout the body [9] and we have recently shown that functional MR1 is expressed in the brain [14]. Moreover, its level directly correlates with a poor outcome in MS and glioma [13, 15].

Here, we investigated a potential role for the MR1/MAIT cell axis in AD pathogenesis. We report that increased levels of MR1 and the number of MAIT cells in the brain are associated with the extent of Aβ plaque pathology. Notably, the lack of the MR1/MAIT cell axis in mice on the 5XFAD AD background delays the development of this pathology.

## Materials and methods

### Human brain samples

Deidentified brain sections were provided by the Indiana Alzheimer’s Disease Research Center from individuals who had either no diagnosis of neurodegenerative disease (non-AD tissue) or individuals who were confirmed to have AD after death.

### Human tissue immunofluorescence (IF)

IF was done with free-floating sections as previously described [16]. Sections were washed in 1X phosphate-buffered saline (PBS) + 0.1% Triton (1XPBS-T) for 20 min followed by 3 h in 1XPBS + 3% Triton (1XPBS-3%T) at room temperature (RT). Next, tissues were incubated in immuno buffer [5% Goat Serum (0060-01, SouthernBiotech, Birmingham, AL), 10% Bovine Serum Albumin (BSA) (A7030, Sigma, St. Louis, MO), 10% Sodium Azide (S2002, Sigma), and 1XPBS + 0.3% Triton] overnight on a rotating platform at 4 °C. Primary antibodies: rabbit anti-IBA1 (polyclonal, 1:50; 019-19741, Wako Chemicals, Richmond, VA), mouse anti-amyloid beta (monoclonal, 1:50, NBP2-23075, Novus Bio, Centennial, CO), and mouse anti-MR1 (monoclonal, 1:50, 361102, Biolegend, San Diego, CA) were added and incubated for 7 days on a rotating platform at 4 °C. Following primary incubation sections were washed in 1XPBS-T and then were incubated in secondary antibodies: DAPI (62248, Thermofisher, Waltham, MA), goat anti-rabbit IgG Alexa fluor 568, goat anti-mouse IgG2b Alexa fluor 488, and goat anti-mouse IgG2a Alexa Fluor 647 (1:5000; A11011, A21241, A21141, respectively, Life Technologies, Bartlesville, OK, USA) for 3 days at 4 °C. Tissue sections were washed in 1XPBS-T and mounted on glass slides using Vectashield Antifade (H-1000, Vector Labs, Newark, CA).

### Animals and ethics statements

The 5XFAD mouse model of AD was used throughout the study as the expression of human transgenes, amyloid precursor protein (APP) and presenilin 1 (PSEN1), recapitulate many of the phenotypes of the AD pathology at a quick and progressive manner [17, 18]. 5XFAD mice on the C57BL/6 background were provided by Dr. Gary Landreth (IUSM). MR1-deficient (MR1 KO) mice [14], were kindly provided by Dr. Daniel Hoft (St. Louis University, St. Louis, MO). The 5XFAD mice were bred and crossed onto the MR1 KO mice to produce 5XFAD/MR1 KO mice. The creation of these mice did not have an effect on the expression of the transgenes, APP and PSEN1, in the brain (Additional file 1: Fig. S1A, B). An equal number of males and females was used for each experiment. The mice were bred and housed at the Indiana University School of Medicine. All animal procedures were approved by the Indiana University School of Medicine’s Institutional Animal Care and Use Committee.

### RNA isolation and quantitative, real-time PCR (qPCR)

Mice were euthanized via CO_2_ asphyxiation and perfused with 1XPBS. Brains were sub-dissected and homogenized. RNA was extracted from homogenized tissue using PureLink RNA Mini Columns following the manufacturer's instructions. RNA was reverse-transcribed into cDNA using the High-Capacity RNA to cDNA set. Taqman, MasterMix, and StepOne Plus (Applied Biosystems, Waltham, MA) were used for qPCR as per the manufacturer's instructions. For the normalization of MR1 (4331182, Thermofisher), the housekeeping gene *Gapdh* was used. The results are presented as the relative fold change in gene expression normalized to the wild-type calibrator. For statistics, the relative ΔCt method was used [19].

### Mouse immunofluorescence (IF)

Mice were euthanized by CO_2_ asphyxiation and perfused with 1XPBS. Brains were removed and post-fixed in 4% paraformaldehyde (PFA) then incubated in sucrose and frozen. IF in mouse free-floating tissue sections was performed similar to the human tissue IF protocol. Sections were washed in 1XPBS-T for 5 min followed by 30 min in 1XPBS-3%T at RT. Next, tissues were incubated in mouse Fc Blocker (5553242, BD Pharmingen, Plainfield, IN) for 1 h followed by goat immuno buffer for 1 h. Primary antibodies: rabbit anti-IBA1, mouse anti-amyloid beta, and mouse anti-MR1 were added as above and incubated for 2 days on a rotating platform at 4 °C. Following primary incubation, sections were washed in 1XPBS-T and then incubated with the same secondary antibodies as above for 1–2 h. Tissue sections were washed in 1XPBS-T and mounted on glass slides using Vectashield Antifade (H-1000, Vector Labs).

### Mouse ThioS and MR1 tetramer IF staining

ThioS staining followed a previously described protocol [20]. IF was performed in mouse free-floating tissue sections. Sections were incubated in 0.1% ThioS (T1892, Sigma), then washed in 70% ethanol, and finally washed in 1XPBS each for 5 min [20]. Next, sections followed the mouse IF protocol with changes to the length of time at each step for staining with the MR1 tetramer. Tissues were incubated in mouse Fc Blocker (5553242, BD Pharmingen, Franklin Lakes, NJ) for 2 h followed by goat immuno buffer for 24 h. 5-OP-RU-loaded MR1 Tetramers (APC-conjugated, NIH Tetramer Core) were added and incubated for 3 days on a rotating platform at 4 °C. Following primary incubation, sections were washed in 1XPBS-T and then incubated with Dapi for 30 min. Tissue sections were washed in 1XPBS-T and mounted on glass slides using Vectashield Antifade (H-1000, Vector Labs). 6-FP-loaded MR1 Tetramers (APC-conjugated, NIH Tetramer Core) served as the negative control.

### Microscopy and semi-quantitative IF densitometry analysis

The IF signal for both human and mouse tissue was visualized using a Nikon Eclipse Ti Inverted microscope with plan fluor 20 × /1.30 and 100 × /1.49 objectives. Images were reconstructed with the NIS Elements v4.5 software (Nikon Instruments). The relative mean pixel intensity of the immunostaining signal was acquired using the Image J Fiji software package V1.53 (NIH) as previously described [21]. Images were converted to black and white, and the entire image density was measured. The background—determined by any area, where the signal was not located—was measured and subtracted from the image density. Any sections that had damage to the area of interest were excluded. For region of interest (ROI) analysis, the selection tool was used to outline the cell boundary and the densitometry was measured in that area.

### Flow cytometry

Mice were anesthetized by CO_2_ asphyxiation and perfused with ice cold 1XPBS. Brains, spleens, and thymi were removed. Brains were passed through a 70-µm nylon cell strainer and centrifuged. Cells were washed in 1XPBS then placed upon a 40% Percoll gradient and centrifuged at 2000 rpm for 20 min at RT. Spleens and thymi were homogenized and centrifuged. All cells from brain, spleen, and thymus followed the rest of the protocol. The cells were incubated in mouse red blood cells lysis buffer for 5 min then centrifuged at 1200 rpm for 5 min. The cells were resuspended in buffer containing the Fcγ receptor-specific blocker, anti-CD16/32 mAb, 2.4G2 (the hybridoma was a kind gift from Dr. J. Yewdell, NIH), then the primary antibodies: anti-TCR-β (PE-labeled; Clone H57–597, 553172, BD Pharmingen), anti-TCR-β (FITC-labeled; Clone H57–597, 109206, Biolegend), CD44 (Pacific Blue-labeled, Clone IM7, 103020, Biolegend), 5-OP-RU-loaded MR1 Tetramer (APC-conjugated, NIH Tetramer Core), CD69 (PE-labeled, Clone H1.2F3, 553237, BD Pharmingen), CD25 (FITC-labeled; Clone 7D4, 553071, BD Pharmingen), B220 (PerCP-Cy5.5-labeled; Clone: RA3-6B2, 552771, BD Pharmingen), and MR1 (APC-labeled; Clone: 26.5, 361108, Biolegend) were added depending upon the experiment and kept on ice for 30 min. Next, the cells were washed and fixed in 1% PFA, then washed in 1XPBS and FACS buffer (1% BSA, 0.1% Sodium Azide, and 1XPBS). All data were acquired using a BD LSRFortessa™ cell analyzer (BD Bioscience, Franklin Lakes, NJ) and analyzed using FlowJo v10 software (Becton Dickinson, San Jose, CA). Cells stained with an isotype control mAb and 6-FP-loaded MR1 tetramers served as our negative controls.

### Statistical analysis

**Fig. 1:**
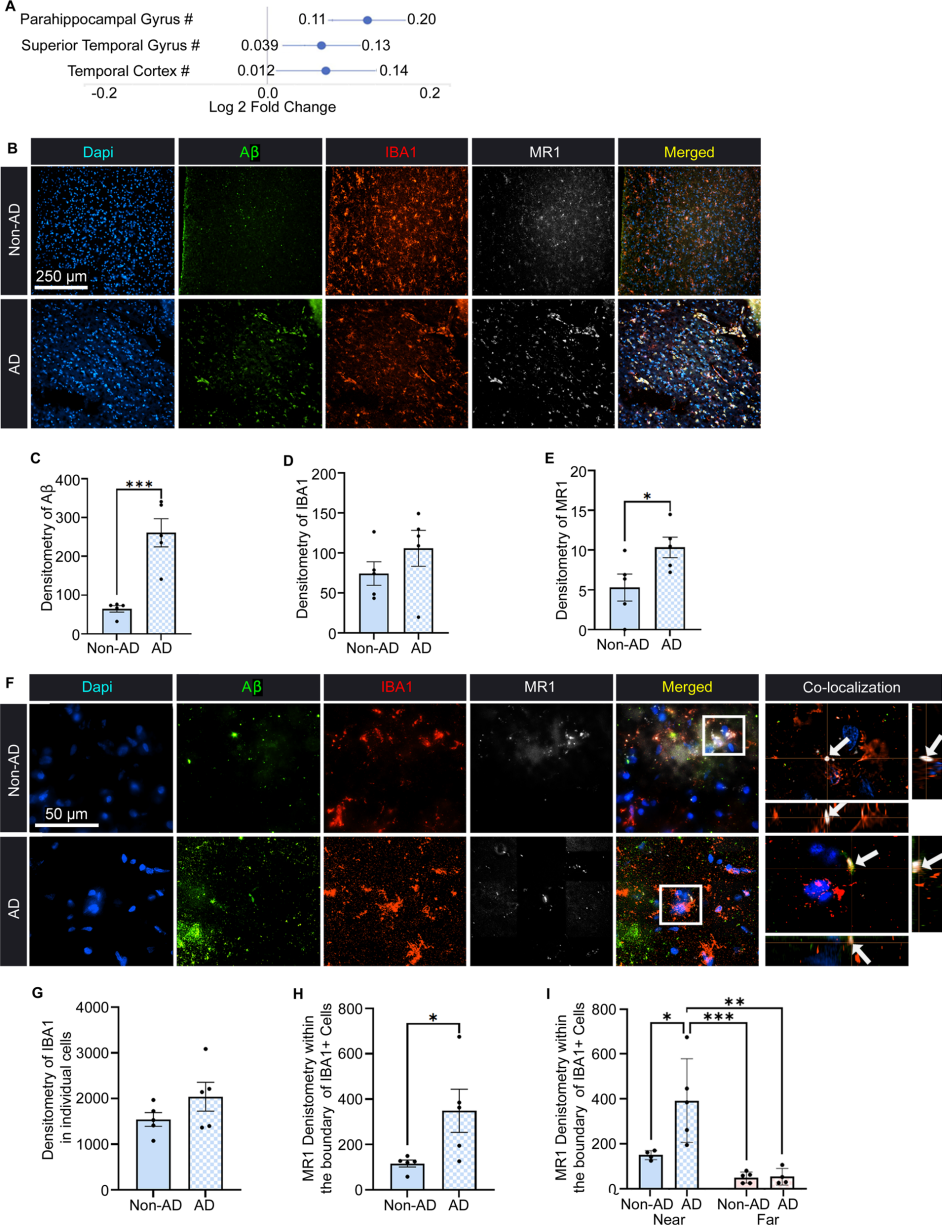
Immunostaining of microglia, amyloid-beta (Aβ), and MR1 in the temporal cortex of individuals with Alzheimer’s disease (AD). **A** Fold change in *MR1* gene expression in human AD from the Agora Database. **B** Representative images of AD and non-AD control tissue showing Dapi (blue), Aβ (green), IBA1-labeled microglia/macrophages (red), MR1 (white), and these images merged. Scale bar = 250 µm. **C–E** Densitometry analysis presented as the relative mean pixel intensity for Aβ (**C**), IBA1 (**D**), and MR1 (**E**). **F** Representative images of non-AD and AD tissue at 100 × with co-localization of the boxed regions in the XY, ZX, and ZY planes (arrows). Scale bar = 50 µm. **G**, **H** Densitometry analysis presented as the relative mean pixel intensity for IBA1 (**G**) and MR1 expression within the boundaries of the IBA1 + cells (**H**). **I** Densitometry analysis of the MR1 signal within the boundaries of the IBA1 + cells that are determined to be near (visibly touching) or far from Aβ plaques (no visible touching). Statistical analysis was performed using a Student’s *t* test (**C**–**E**, **G**–**I**) and a two-way ANOVA with Tukey post hoc multiple comparison test (**I**). *P < 0.05, **P < 0.01, ***P < 0.001 (*n* = 5/group). Significance in both males and females was normalized to the age of death. Data are shown as the mean ± standard error of the mean

**Fig. 2:**
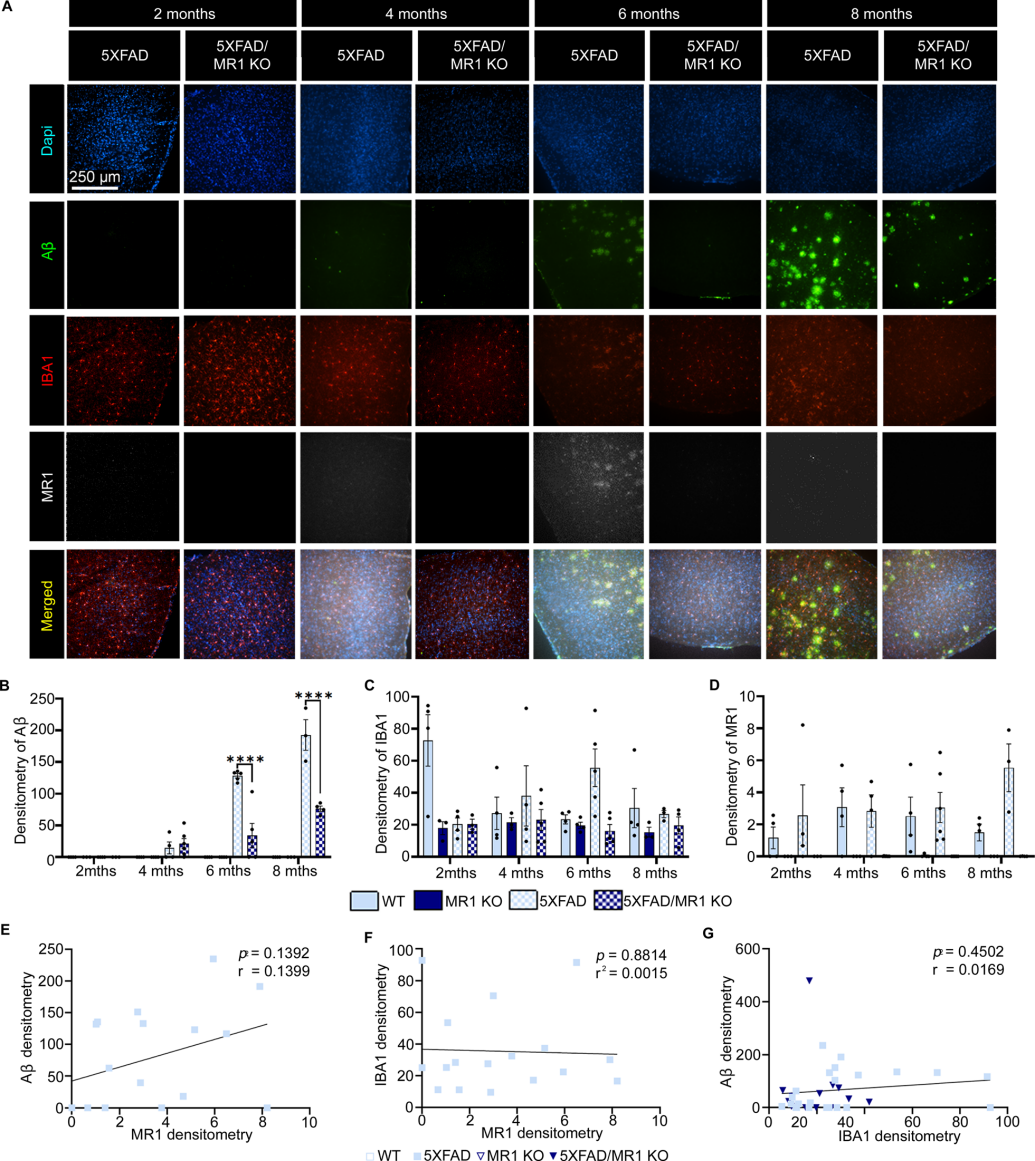
Densitometry analysis of microglia, amyloid-beta (Aβ) plaques, and MR1 in the temporal cortex of 5XFAD mice. **A** Representative images highlighting amyloid beta pathology showing Dapi (blue), IBA1-labeled microglia/macrophages (red), Aβ (green), MR1 (white), and the images merged. Scale bar = 250 µm. **B**–**D** Densitometry analysis as the relative mean pixel intensity for Aβ (**B**), IBA1 (**C**), and MR1 (**D**). **E**–**G** Graphs for the correlational analysis of the densitometry results between MR1 and Aβ compare 5XFAD (squares) and 5XFAD/MR1 KO mice (upside-down triangles) (**E**), MR1 and IBA1 (**F**), and IBA1 and Aβ (**G**). Statistical analysis was performed using a two-way ANOVA with Tukey post hoc multiple comparison test (**B**–**D**) or a simple linear regression analysis (**E**–**G**). ****P < 0.0001 (**B**–**D**: *n* = 3–7/group; **E**–**G**: *n* = 17–21/group). The data are shown as the mean ± standard error of the mean

**Fig. 3:**
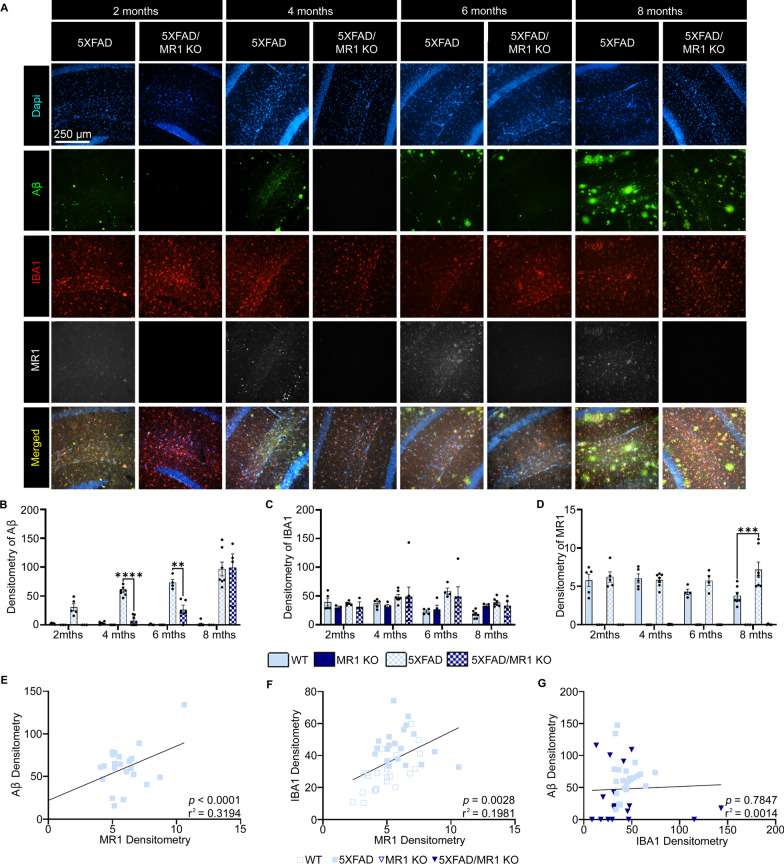
Densitometry analysis of microglia, amyloid-beta (Aβ) plaques, and MR1 in the CA1 region of the hippocampus in 5XFAD mice. **A** Representative images highlighting amyloid beta pathology shows Dapi (blue), IBA1-labeled microglia/macrophages (red), Aβ (green), MR1 (white), and the images merged. Scale bar = 250 µm. **B**–**D** Densitometry analysis presented as the relative mean pixel intensity for Aβ (**B**), IBA1 (**C**), and MR1 (**D**). **E**, **F** Graphs for the correlational analysis of the densitometry results between MR1 and Aβ show 5XFAD (squares) and 5XFAD/MR1 KO (upside-down triangles) (**E**), MR1 and IBA1 (**F**), and IBA1 and Aβ (**G**). Statistical analysis was performed using a two-way ANOVA with Tukey post hoc multiple comparison test (**B**–**D**) or a simple linear regression analysis (**E**–**G**). **P < 0.01, ***P < 0.001, ****P < 0.0001 (**B**–**D**: *n* = 4–6/group; **E**–**G**: *n* = 17–21/group). The data are presented as the mean ± standard error of the mean

**Fig. 4:**
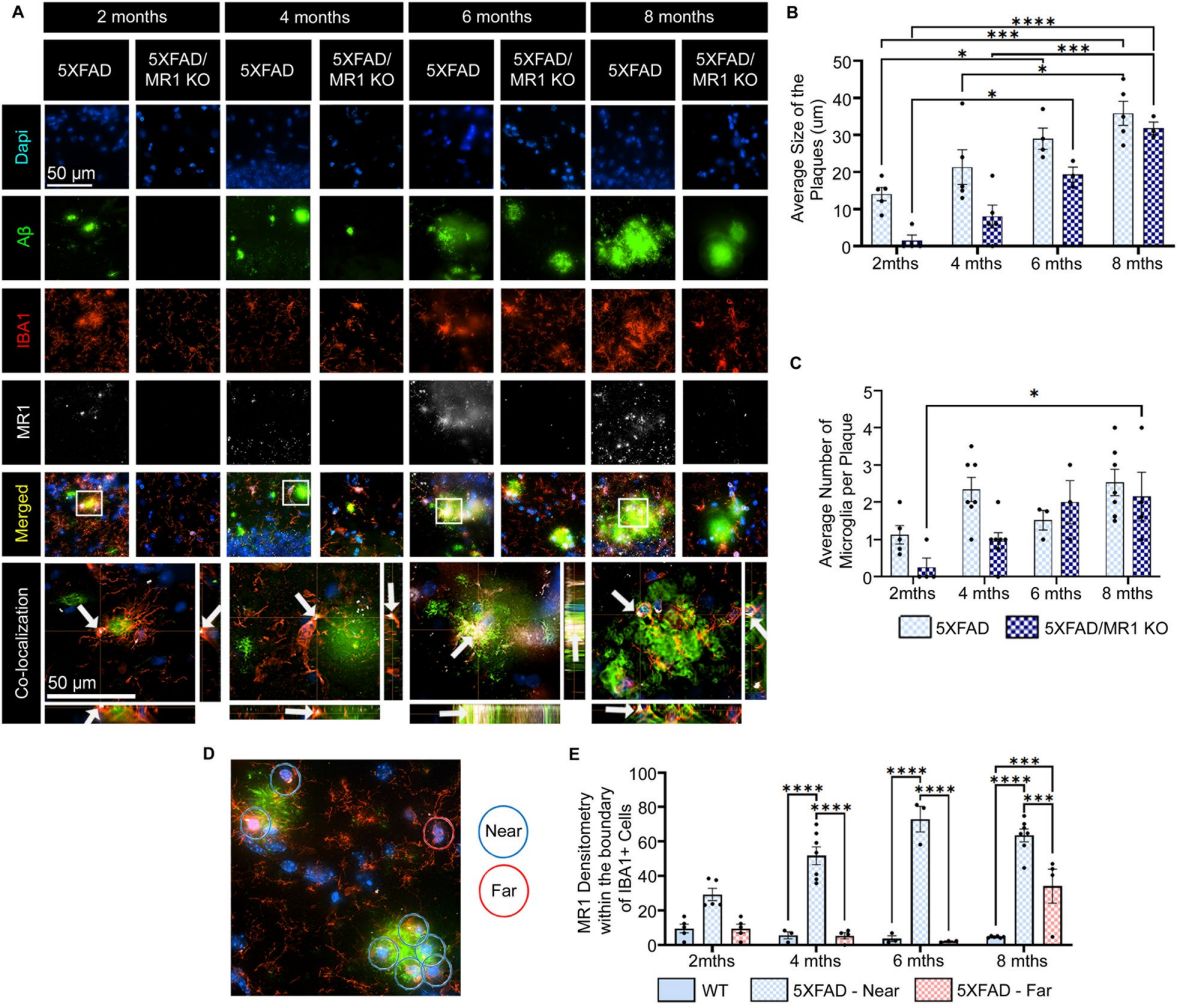
Temporal analysis of amyloid-beta (Aβ) plaques and microglia in 5XFAD vs. 5XFAD/MR1 KO mice. **A** Representative images showing Dapi (blue), IBA1-labeled microglia/macrophages (red), Aβ (green), MR1 (white), and the images merged, with co-localization of the boxed regions in the XY, ZX, and ZY planes (arrows). Scale bar = 50 µm. **B** The average diameter of Aβ plaques. **C** Average number of microglia visibly touching the Aβ plaques. **D** Representative image showing how microglia were determined to be near (blue circle) or far from plaques (red circle). **E** Densitometry analysis of the MR1 signal within the boundaries of the IBA1+ cells that were determined to be near (visibly touching) or far from Aβ plaques (no visible touching). Statistical analysis was performed using a two-way ANOVA with Tukey post hoc multiple comparison test. *P < 0.05, ***P < 0.001, ****P < 0.0001 (*n* = 3–7/group). The data are shown as the mean ± standard error of the mean

**Fig. 5:**
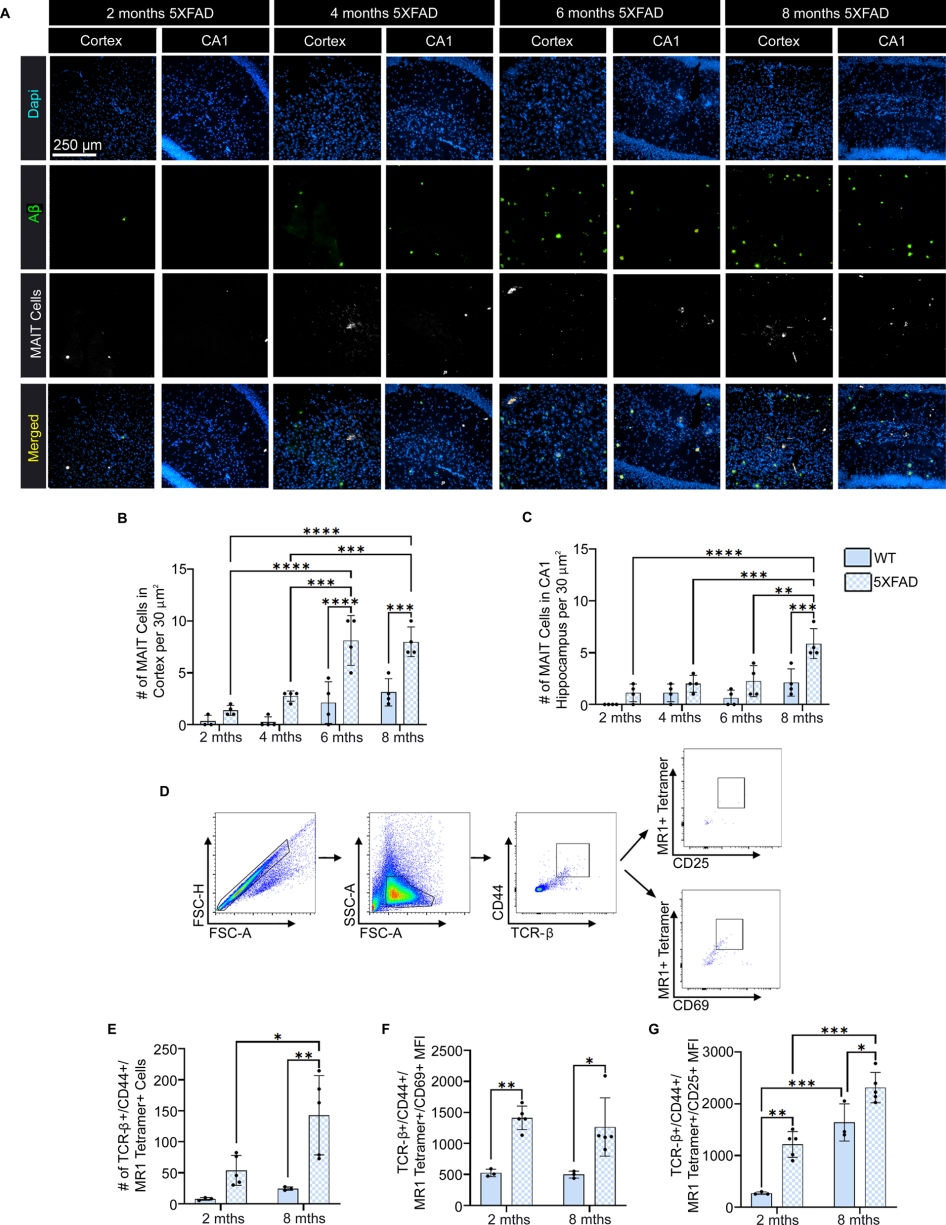
Temporal changes in the number of mucosal-associated invariant T (MAIT) cells in the brains of 5XFAD mice and their activation state. **A** Representative images showing Dapi (blue), amyloid-beta (Aβ) (green), MAIT cells (white), and the images merged. Scale bar = 250 µm. **B, C** Quantification of the number of MAIT cells in the temporal cortex (**B**) and CA1 region of the hippocampus (**C**). **D** Representative flow cytometry panels showing the gating strategy for the identification of MAIT cells (TCRβ+ CD44+ MR1 Tetramer+) and measurement of their activation state (CD69+ or CD25+). **E** Number of MAIT cells in the brain at 2 and 8 months of age. **F**, **G** Mean fluorescence intensity (MFI) of CD69 (**F**) and CD25 (**G**) expression on MAIT cells. Statistical analysis was performed using a two-way ANOVA with Tukey post hoc multiple comparison test. *P < 0.05, **P < 0.01, ***P < 0.001, ****P < 0.0001 (*n* = 3–4/group). The data are shown as the mean ± standard error of the mean

Graphpad Prism software v9.4 was used for statistical analyses. All samples were blinded to the researcher before the start of each analysis. Student’s *t* test was used to analyze human tissue and mouse qPCR in Fig. 1C–E, G, and H and Additional file 1: Fig. S1. A two-way ANOVA with Tukey’s multiple comparisons test was used to analyze the multiple comparisons of Figs. 1I, 2B–D, 3B–D, 4B, C and E, 5B, C and E–G and Additional file 1: Figs. S2C, D, and 4B, C. Simple linear regression was used to determine correlational analysis for Figs. 2E–G and 3E–G. All statistical tests were two-tailed with an alpha of 0.05. All data are shown as the mean ± standard error of the mean (SEM). All figures were made in Adobe Photoshop Elements 13.

## Results

### Correlation between microglial MR1 expression and pathology in human AD brain tissue

We have recently reported that the overexpression of MR1 correlates with a worse prognosis in patients with glioma [15]. Moreover, elevations in MR1 levels have been detected in CNS lesions of MS patients [13]. Interestingly, in accessing the Agora AD database which was generated from over 1100 individuals [22, 23], we found that MR1 gene expression is increased in the temporal cortex, parahippocampal gyrus, and superior temporal gyrus in AD patients normalized to the age of death (Fig. 1A). Those regions are highly susceptible to AD pathology and are involved in memory [24, 25]. The next step was to determine if this was also reflected in MR1 protein levels by immunofluorescent microscopy. Due to limited availability of AD patient brain tissue and a focus on direct comparisons between humans and mice, only the temporal cortex was analyzed as the other regions do not have an exact structure in the mouse [26, 27]. The temporal cortical tissue samples used in this study were obtained from five deceased patients who were diagnosed with AD or from five individuals who had no neurodegenerative disease diagnosis at the time of death. These tissues were then stained with antibodies against MR1, microglia/macrophages (IBA1), and Aβ (Fig. 1B). We used densitometry analysis to quantify the immunofluorescent images. The AD group had significantly increased levels of Aβ [*t*(8) = 5.251; *P* = 0.0008] (Fig. 1C), with no change in IBA1 densitometry between the groups [*t*(8) = 1.172; *P* = 0.2749] (Fig. 1D). Remarkably, there was a significant increase in microglial MR1 expression in AD patients as compared to non-AD controls [*t*(8) = 2.363; *P* = 0.0458] (Fig. 1E). Next, we assessed the individual microglia at 100 × magnification and analyzed both IBA1 density of the microglia (Fig. 1G) and the level of intracellular and cell surface MR1 present, based on microglial distance from plaques (Fig. 1H). Although IBA1 density was no different between the groups [*t*(8) = 1.422; *P* = 0.1927], there was a significant increase in the average MR1 signal present inside the microglial boundary in the AD group [*t*(8) = 2.414; *P* = 0.0422]. We further explored the intensity of the intracellular and cell surface MR1 signal based on whether microglia were near and touching plaques, or if they were far and not visibly touching plaques (Fig. 1I). Interestingly, there was a higher level of MR1 expression in those microglia closer to plaques; those IBA1+ cells further away from the plaques had lower MR1 levels. Moreover, there was a significantly greater overall level of MR1 in AD patient microglia as compared to non-AD controls [*F*(1,23) = 6.119; *P* = 0.0268]. Together, these data suggest that intracellular and cell surface MR1 levels increase when microglia are closer to plaques than further away and could thereby serve as another marker to define AD plaque pathology.

### Importance of MR1 expression in the 5XFAD mouse model of AD

Having found that MR1 expression was increased in microglia that are in close proximity to plaques in AD patient temporal cortex tissue, we wanted to see if the same occurs in the 5XFAD mouse model of AD [3]. First, we performed qPCR on the temporal cortex of 8-month-old 5XFAD mice as compared to age- and sex-matched control C57BL/6 wildtype (WT) mice to determine if *Mr1* gene expression levels were higher in the AD mice vs. controls. As we found in data mined from the Agora database in human AD tissue, *Mr1* mRNA transcript levels were also significantly higher in 5XFAD as compared to WT control mice [*t*(14) = 2.961; *P* = 0.0103] (Additional file 1: Fig. S1C). This gave us confidence that the 5XFAD model would be very useful in dissecting the importance of MR1 expression in the development of AD pathology.

### MR1-deficient 5XFAD mice have a significantly reduced plaque burden compared to wildtype 5XFAD

To understand how microglial MR1 expression may be altering Aβ plaque pathology, we performed immunofluorescent microscopy analyses of brain sections from MR1-deficient (MR1 KO) mice crossed onto the 5XFAD background (5XFAD/MR1 KO) (Fig. 2 and Additional file 1: Fig. S2). We then analyzed these mice at four distinct timepoints, where the 5XFAD model has been shown to begin the development of detectable plaque formation and gliosis (2 months), synaptic loss and memory deficits (4 months), neuronal loss (6 months), and all the major pathologies together (8 months) [28]. In the temporal cortex, as expected, there was no Aβ in control WT mice. In 2–4-month-old 5XFAD mice, there was no difference in the levels of Aβ compared to 5XFAD/MR1 KO (*P* > 0.9999). In striking contrast, 6- and 8-month-old 5XFAD mice had significantly increased Aβ compared to 5XFAD/MR1 KO (8 months: [*F*(9,48) = 23.6; *P* < 0.0001]) (Fig. 2B). In terms of IBA1 densitometry, although altered across the different genotypes [*F*(9,46) = 2.983; *P* = 0.0070] (Fig. 2C), there was no significant direct change. Neither was there a significant change in MR1 signal between control WT mice and 5XFAD [*F*(9,48) = 0.9023; *P* = 0.5307] (Fig. 2D); there was no MR1 signal in the MR1 KO mice [*F*(3,48) = 15.96; *P* < 0.0001]. To further understand if MR1 is related to the IBA1 or Aβ signals, we analyzed the direction of the relationship between MR1, microglia, and Aβ plaques. Linear regression was performed to look for correlations between the data. There was no significant correlation between MR1 and Aβ (*r*^2^ = 0.1399; *P* = 0.1392) (Fig. 2E), MR1 and IBA1 (*r*^2^ = 0.0015; *P* = 0.8814) (Fig. 2F), or Aβ and IBA1 (*r*^2^ = 0.0169; *P* = 0.4502) (Fig. 2G), indicating no relationship between these variables in the cortex.

The temporal cortex is a part of the intricate circuit involving memory [29–31]. We analyzed another part of this circuit to determine if AD pathology development in the temporal cortex is unique or if other regions showed a similar response to the lack of MR1. The CA1 region of the hippocampus in 5XFAD mice was examined (Fig. 3 and Additional file 1: Fig. S3), as it has been shown to be one of the most susceptible regions of the brain in AD [29, 30]. Using densitometry analysis of the immunofluorescence images, the levels of Aβ were found to be lower in the absence of MR1 at the early timepoints [*F*(9, 61) = 8.811; *P* < 0.0001] (Fig. 3A). For example, 5XFAD mice had a significantly higher Aβ density than the 5XFAD/MR1 KO groups (*P* < 0.0001). Even at 6 months, the amount of Aβ in the 5XFAD group was still higher than that in 5XFAD/MR1 KO mice (*P* = 0.006). There was no change in the overall level of IBA1 in this hippocampal region [*F*(9, 61) = 0.8400; *P* = 0.5826] (Fig. 3C). As expected, no MR1 signal was detected in the MR1 KO mice [*F*(9, 61) = 130.5; *P* < 0.0001] (Fig. 3D). MR1 levels in microglia from control WT mice remained at a comparable level as 5XFAD mice until 8 months of age, when the MR1 signal in 5XFAD mice was elevated (*P* = 0.0009). To ensure that this increase in microglial MR1 was due to the Aβ plaque pathology rather than the 5XFAD transgenes, MR1 expression was analyzed on another cell type that has high endogenous levels of MR1—peripheral B cells in both thymus and spleen, which are two main peripheral lymphoid organs (Additional file 1: Fig. S4). In both tissues, B cell MR1 expression was not altered between 5XFAD and control mice, but only increased as a result of aging [Thymus: *F*(1,12) = 0.0261; *P* = 0.8744; Spleen: *F*(1,12) = 0.0011; *P* = 0.9741]. Together, these data indicate that in 5XFAD mice that lack MR1, amyloid build-up in the early stages is significantly delayed, especially in the CA1 region. To determine if, unlike in the cortex, there was a directional relationship between MR1, microglia, and Aβ plaques in the CA1 region, a linear regression analysis was used. MR1 levels were positively correlated with Aβ densitometry (*r*^2^ = 0.3194; *P* < 0.0001) (Fig. 3E). Thus, as MR1 increased, there was a concomitant increase in Aβ plaque density. With IBA1, a similar positive correlation with MR1 densitometry was found (*r*^2^ = 0.1981; *P* = 0.0028) (Fig. 3F). However, when analyzing IBA1 and Aβ, we found that there was no correlation in their respective densities (*r*^2^ = 0.0014; *P* = 0.7847) (Fig. 3G). Interestingly, however, when 5XFAD/MR1 KO mice were excluded from the analysis, we did find a significant correlation between the densities of IBA1 and Aβ in control WT vs. 5XFAD mice (*r*^2^ = 0.1562; *P* = 0.0128). This further indicates that the KO mice do not follow a typical response. Together, this suggests that in the CA1 region of the hippocampus, MR1 is highly involved in the interaction between microglia and Aβ.

### Reduced amyloid-beta plaque size and numbers in MR1-deficient 5XFAD mice

To further analyze the relationship between MR1, microglia, and Aβ plaques, the diameters of the plaques were analyzed to determine if an MR1 deficiency had an impact on the overall size of the plaques. We also counted the number of IBA1+ cells visibly touching Aβ plaques to determine if the microglial response was altered in the 5XFAD/MR1 KO mice in the CA1 region of the hippocampus (Fig. 4). Plaques in the 5XFAD group had an overall larger diameter than those in 5XFAD/MR1 KO mice [*F*(1,26) = 19.23; *P* = 0.0002] (Fig. 4B), although the diameter of the plaques in both groups increased over time [*F*(3,26) = 25.34; *P* < 0.0001]. When examining microglia from 5 and 5XFAD/MR1 KO mice that were physically interacting with Aβ plaques, there was no change in their number between the groups [*F*(3,32) = 2.027; *P* = 0.1299] (Fig. 4C). There was, however, an increase in the number of microglia visibly touching the plaques across time in both groups [*F*(3,32) = 6.586; *P* = 0.0014]. Taken together, these data indicate that a lack of MR1 does not impair the ability of microglia to interact with plaques; rather, an MR1-deficient environment reduces plaque number and overall pathology in the brain.

Although there was no observable change in the ability of 5XFAD/MR1 KO microglia to interact with Aβ plaques, it was nonetheless important to determine whether MR1 has a role in that interaction. Similar to the analysis of human tissue displayed in Fig. 1I, MR1 expression within and on the microglia that were near vs. far from Aβ was determined in 5XFAD mice (Fig. 4D). There was a significant upregulation in MR1 expression within the boundaries of the IBA1+ cells near the Aβ plaques [*F*(6,43) = 9.207; *P* < 0.0001] (Fig. 4C). By 8 months, MR1 expression was even increased in IBA1+ cells located far from plaques (*P* = 0.0009); however, this could have been due to the difficulty of finding IBA1+ cells not near Aβ plaques. Therefore, these data suggest that, overall, the closer microglia are to a plaque, the greater their level of intracellular and cell surface MR1.

### MAIT cells in the brains of 5XFAD mice are significantly increased and highly activated

After finding increased MR1 expression in 5XFAD mice and reduced Aβ pathology in 5XFAD/MR1 KO mice that lack the MR1/MAIT cell axis, we investigated the contribution of MAIT cells to the AD pathology. MAIT cells are the prototypic MR1-restricted T cell subpopulation [32, 33] and can easily be identified using MR1 tetramers [14, 34, 35]. Although MAIT cells are quite abundant in human peripheral blood (1–10% of circulating T cells [36]), they are much less so in mice. Thus, identifying the location of these MAIT cells can be challenging, with MR1 tetramers only recently being used for histology [34, 35]. The number of MAIT cells was assessed in the cortex and CA1 region of the hippocampus during the progression of AD (Fig. 5). An MR1 tetramer that does not stain MAIT cells was used as a negative control to confirm the specificity of the signal (Additional file 1: Fig. S5). Strikingly, there was a significant temporal increase in the number of MAIT cells in the cortex, with 6- and 8-month-old 5XFAD mice having an increased number of MAIT cells per area compared to control mice and those at the earlier timepoints [*F*(3,23) = 23.74; *P* < 0.0001] (Fig. 5B). In the CA1 region of the hippocampus, only the 8-month timepoint in the 5XFAD mice had a significantly increased number of MAIT cells relative to controls and the earlier 5XFAD timepoints [*F*(3,24) = 15.95; *P* < 0.0001] (Fig. 5C). These data indicate that MAIT cells are increased in the brain, concomitant with the extent of Aβ plaque pathology. To further understand how these MAIT cells may be responding in the brain, we studied the activation state of the MAIT cell using the classical T cell activation markers CD69 and the IL-2 receptor α chain, CD25. Due to the technical inability to co-stain with MR1 tetramers and mAbs in tissue sections, we used flow cytometry of whole brain mononuclear cells to determine the activation state of the MAIT cells. We have previously reported that there are brain-resident MAIT cells in the normal mouse brain [14]. Thus, in this experiment, we focused on analyzing the activation state of brain-resident MAIT cells in 5XFAD mice at the earliest timepoint, where MAIT cells are not increased (i.e., 2 months of age) and the oldest timepoint (8 months), where we found an increase in MAIT cells in both the cortex and hippocampal CA1 region. As expected, in our flow cytometry analyses, there was an increased number of MAIT cells in 5XFAD mouse brains at the 8-month timepoint [*F*(1,12) = 16.34; *P* = 0.0016] (Fig. 5E). Within this population of MAIT cells, there was an enhanced level of CD69 expression at both 2 and 8 months in the 5XFAD mice as compared to controls [*F*(1,12) = 29.61; *P* = 0.0001] (Fig. 5F), suggesting that there was an elevated level of MAIT cell activation in these mice. Furthermore, this population also had increased expression of CD25 at both the 2- and 8-month timepoints as compared to controls [*F*(1,12) = 34.46; *P* < 0.0001] (Fig. 5G). Interestingly, in the 5XFAD mice, there was a significantly higher expression level of CD25 on MAIT cells at 8 months as compared to those at 2 months [*F*(1,12) = 80.15; *P* < 0.0001]. Of important note, this increase in these activation markers was only found in MAIT cells and not in any other non-MAIT T cells [CD69: *F*(1,12) = 1.01; *P* = 0.3346; CD25: *F*(1,12) = 0.9653; *P* = 0.3452] (Additional file 1: Fig. S6A, B). Therefore, these data strongly suggest that the development of AD pathology impacts the number and activation state of MAIT cells in the brain.

## Discussion

Alzheimer’s disease is a devastating neurodegenerative disease that is growing as a worldwide epidemic, mainly affecting the elderly [1, 2]. Although neuroinflammation facilitates a significant amount of brain pathology in AD, there is still very little known about how the immune system plays a role [2, 37, 38]. This study aimed to understand the role of the MHC class I-like MR1 molecule and MAIT cells in AD pathology. Upon mining the Agora data set, we found MR1 gene expression was significantly elevated in the temporal cortex and parahippocampal gyrus in AD patients. Focusing our studies in tissues from AD patients and the 5XFAD mouse model that either did (or did not) express MR1 or have MAIT cells, it was clear that the MR1/MAIT cell axis contributes to the temporal development of AD pathology. Our observations can be broken down into four main themes:

### Gene and protein expression of MR1 is elevated in brain tissue from AD patients and 5XFAD mice

This is the first study to demonstrate that not only is *MR1* gene expression increased (Agora Database) but there is an increase in MR1 protein expression in human AD tissue. This is crucial, as it shows that not only are the levels of MR1 upregulated in AD, but increased MR1 can also be found along the surface of microglia/macrophages in AD. The Agora Database has analyses of over 1100 post-mortem individual AD patient brain samples in different brain regions. Our study used samples from five human brains per group. As such, we focused our analyses on the temporal cortex due to the limited availability of this precious brain tissue. Despite the smaller “n” of the AD patient samples, our findings in 5XFAD mice reported here are in line with evidence found in the Agora database, which strongly supports a role for the MR1/MAIT cell axis in AD pathology development. Furthermore, we confirmed that the 5XFAD mouse model has a comparable enhancement of MR1 gene and protein expression. This is particularly important, as there are a number of issues surrounding animal models of AD and their lack of exactly replicating the disease pathology observed in AD patients [39, 40]. Although the 5XFAD model does not replicate the entire range of AD pathology, it does follow the MR1 expression patterns seen in humans, making it suitable for studying the MR1/MAIT cell axis. Currently, little is known about the MR1/MAIT cell axis in neurological diseases with limited investigation into only five neurological diseases including glioma [15], MS [11, 12, 41], ischemia [42], experimental autoimmune encephalomyelitis [10], and cerebral palsy [43]. In glioma and MS, increased MR1 expression correlates with a worsening prognosis of the disease [13, 15]. Whereas in ischemia, stopping the influx of MAIT cells into the brain reduces symptom severity [42]. For AD, this means that the MR1/MAIT cell axis may have a negative outcome on AD pathology by worsening disease progression.

### MR1 levels in and on microglia are higher in those that are nearer to Aβ plaques and are positively correlated with Aβ levels

Only in MS and cancer has human MR1 expression been investigated in terms of disease severity outcomes. In MS, the expression of MR1 is highly upregulated in lesion areas, regardless of the type of lesion [13]. In breast, renal, thyroid, and lung cancer, as well as glioma, *MR1* gene expression is increased. However, only in glioma does disease severity and overall survival correlate with higher MR1 gene and protein expression [15]. Alongside MS and glioma, our findings in AD show a relatively comparable effect, as elevated MR1 levels are correlated with increased Aβ only in the most vulnerable area—the CA1 region of the hippocampus [29]. In addition, MR1 expression is higher in microglia that are closer to Aβ plaques than those further away, in both human AD patients and 5XFAD mice. Interestingly, another antigen-presenting molecule, MHCII, has also been found to be co-localized with Aβ plaques, although not all microglia that are near plaques are MHCII positive in 5XFAD mice [6]. Of note, similar to monocytes, microglia produce IL-18, which is able to stimulate MAIT cells [44, 45]. In AD, IL-18 levels are increased [46, 47] and this is correlated with elevated *Aβ* production [47]. Enhanced expression of MR1 near lesions in MS, or Aβ plaques in AD, suggests that either MR1 + cells in these areas are trying to recruit MAIT cells to the region or, alternatively, MR1 may have a role within glial cells that aids in how the cells handle these areas. Together, this further supports a role for the MR1/MAIT cell axis in Aβ plaque accumulation.

### A germline deletion of MR1 in 5XFAD mice significantly delays the development of Aβ pathology

Having found associations between MR1 expression and brain pathology in AD patients, we used a validated MR1 KO mouse and crossed this onto the 5XFAD mouse model of AD, to assess the potential contribution of the MR1/MAIT cell axis in AD. Interestingly, in AD, MR1 shows the opposite effect of the better-studied classical antigen-presenting molecule, MHCII. Like MR1, MHCII gene expression is increased in human AD (AGORA database); however, there is a worsening of AD pathology in MHCII-deficient AD mice [6]. Microglia that are high in MHCII show a more phagocytic phenotype compared to microglia that lack MHCII. Furthermore, in MHCII-deficient 5XFAD mice, there is an increase in Aβ plaques, with less microglia co-localizing with Aβ plaques [6]. Notably, MHCI is also increased in human AD (AGORA database), although no studies to date have focused on the role of MHCI in AD. However, in aging, it has been found that increased MHCI expression reduces cognitive performance and synaptic density [8]. These data indicate separate roles for the two main classical antigen-presenting molecules in these disorders; MHCII has a positive role in removing Aβ plaques, whereas MHCI enhances the negative effects of aging. However, it should be noted that MHCII in other CNS diseases contributes to pathology differently. For example, MHCII knockout mice undergoing ischemia have reduced CD4 T cell numbers and inflammation, which improves the ischemic outcomes overall [48]. Similarly, a lack of MR1 reduces the overall severity of ischemia in mice [42]. These studies demonstrate the importance of antigen-presenting molecules and the T cells that recognize them, and this supports the idea that the MR1/MAIT cell axis alters neurological disease progression.

### MAIT cell numbers are increased and highly activated in brains of 5XFAD mice with significant pathology

In several CNS diseases, including MS and ischemic stroke, MAIT cell numbers have been found to be increased [42, 45]. Specifically in MS, not only is there an increase in the number of MAIT cells found in the brains of individuals who have lesions, but MR1 levels are elevated as well [45]. In addition, in MS, there are more MAIT cells in the cerebral spinal fluid, the numbers of which are reduced following treatments that decrease MS symptoms [49]. Likewise, in ischemic stroke, treatment with an MR1 ligand that blocks MAIT cell activation immediately following the event reduces the neurologic severity score as well as the amount of cell death [42]. In MS, there is an increased number of activated MAIT cells [12, 45]. Following treatment to reduce MS symptoms, there is a reduction in the number of MAIT cells found in the blood [12]; this is also found in individuals who are in the remission stage of MS [41]. In this study, we found an increased number of MAIT cells at later stages of pathology and these MAIT cells were in an increased activation state that was not present in other T cells. These activated MAIT cells can produce increased pro-inflammatory cytokines including interferon-gamma (IFN-γ) and IL-17A, which can activate other cells and promote the expression of MHCII [50]. Whereas MHCII has been associated with improved outcomes in AD [6], increased IL-17A levels have recently been found to help lead to the onset of cognitive deficits and synaptic alterations [51], indicating the need to understand in more detail the MR1/MAIT cell axis in AD and how it contributes to pathology overall. Our studies in the current report not only indicate a role for MR1 in AD pathology but also suggests a detrimental role for MAIT cells in AD disease progression.

The MR1/MAIT cell axis having a detrimental effect in AD would follow similar to what has been observed in MS, particularly early in the disease [41, 45, 52]. Based on these results, targeting the MR1/MAIT cell axis could serve as a putative novel therapeutic target. Numerous small organic molecules have been identified as potential MR1 inhibitors that block MAIT cell activation [53]. This adds to the possibility that these drugs, including salicylates and diclofenac, could be used as potential inhibitors for MR1-dependent MAIT cell activation, although these have not yet undergone FDA testing for this purpose [53]. With our results showing that the MR1/MAIT cell axis is detrimental in AD, a logical next step would be an investigation into these drugs as possible treatments. Although the evidence of the MR1/MAIT cell axis being involved in neurological diseases is compelling, to our knowledge, this is the first study that investigates this axis in a neurodegenerative disease. Overall, our study suggests that the MR1/MAIT cell axis plays a major role in the progression of the plaque pathology in AD. Future work is needed to understand the contribution(s) of MR1 and MAIT cells in the entire pathology of AD, including the build-up of tau.

## Supplementary Information

**Additional file 1. **Additional figures.

## Data Availability

The data sets used and/or analyzed during the current study are available from the corresponding author on reasonable request.

## Abbreviations

AD: Alzheimer’s disease
Aβ: Amyloid beta
APP: Amyloid precursor protein
CA1: Cornu ammonis 1
CD25: Interleukin-2 receptor alpha chain
IFN: Interferon
IL: Interleukin
IF: Immunofluorescence
IBA1: Ionized calcium binding adaptor molecule 1
KO: Knockout
MAIT: Mucosal-associated invariant T
MHCI/II: Major histocompatibility complex class I/II
MR1: Major histocompatibility complex class I-related
MS: Multiple sclerosis
NK: Natural killer
PSEN1: Presenilin 1
ROI: Region of interest
Th1: T helper 1
ThioS: Thioflavin S
WT: Wild type
