# Supplementary material for: Control of the temporal development of Alzheimer’s disease pathology by the MR1/MAIT cell axis
Source: J Neuroinflammation. 2023 Mar 21;20:78. doi: 10.1186/s12974-023-02761-6 (PMC10029194; doi:10.1186/s12974-023-02761-6)
Supplement: Supplementary file 1 — Additional file 1. Additional figures. [file 12974_2023_2761_MOESM1_ESM.docx]

**Additional Information**

**for**

**Control of the temporal development of Alzheimer’s disease pathology by the MR1/MAIT cell axis**

Season Wyatt-Johnson et. al

**Figure S1**

**
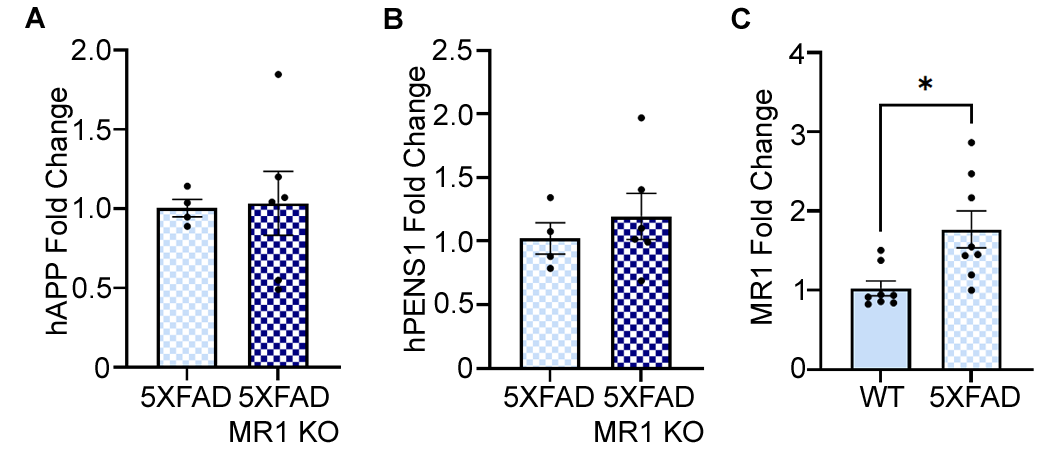
**

**Figure S1.** Temporal cortex qPCR analysis of hAPP and hPENS1 in 5XFAD and 5XFAD/MR1KO mice and fold change of MR1 in 5XFAD mice vs. WT control mice. **A-C** qPCR was performed on RNA isolated from the temporal cortex for hAPP (human amyloid precursor protein) (**A**), hPSEN1 (human presenilin 1) (**B**), and MR1 (**C**). Statistical analysis was performed using Student’s t-test. **P* < 0.05 (*n*=4-8/group). The data are shown as the mean ± standard error of the mean. All mice were 8-months of age.

**Figure S2**


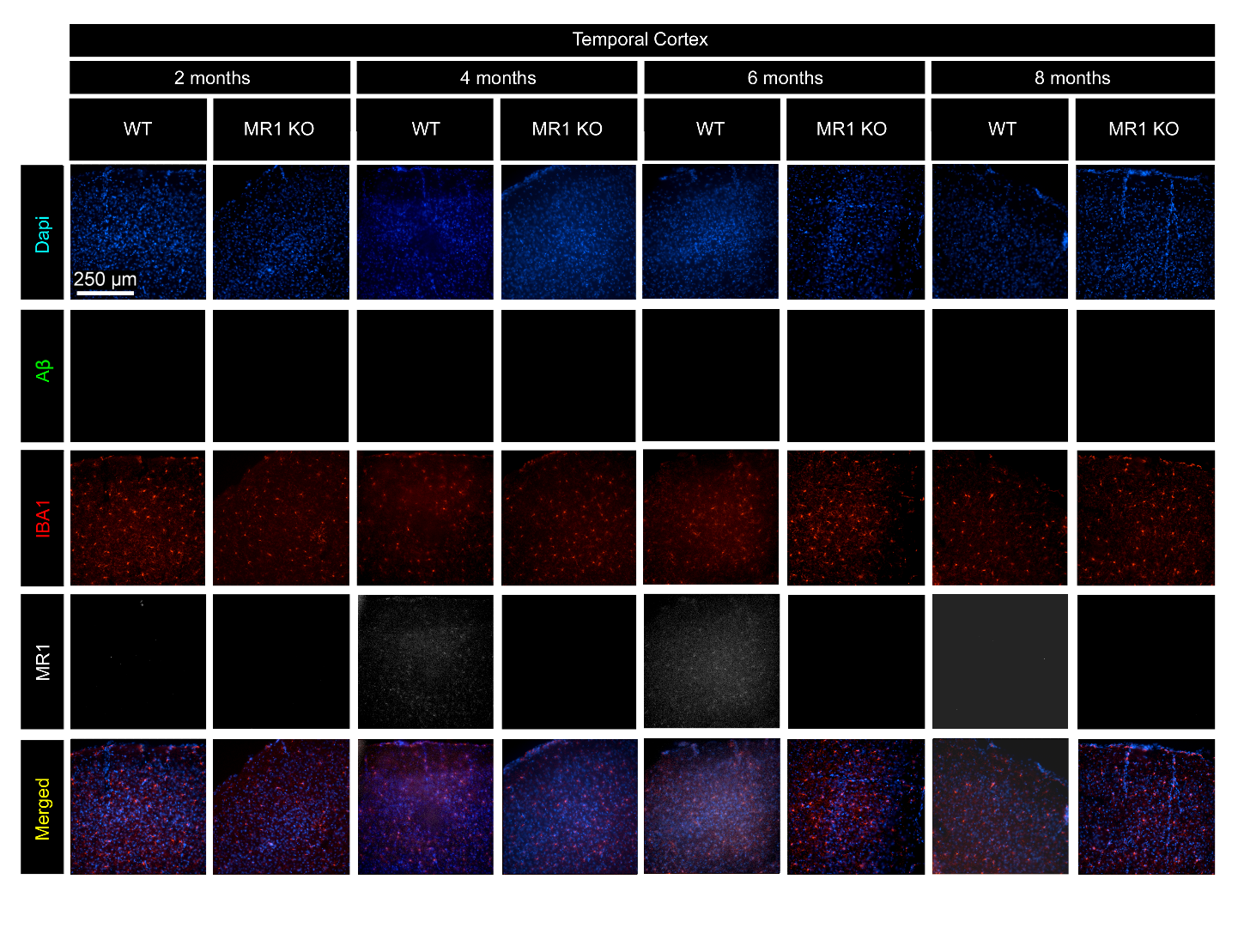
**Figure S2.** Immunofluorescent microscopy of the temporal cortex from wild-type (WT) and MR1 KO mice used for the densitometry analysis shown in Figure 2. Representative images show Dapi (blue), IBA1-labeled microglia/macrophages (red), Aβ (green), MR1 (white) and merged images (**B**). Scale bar = 250 µm.

**Figure S3
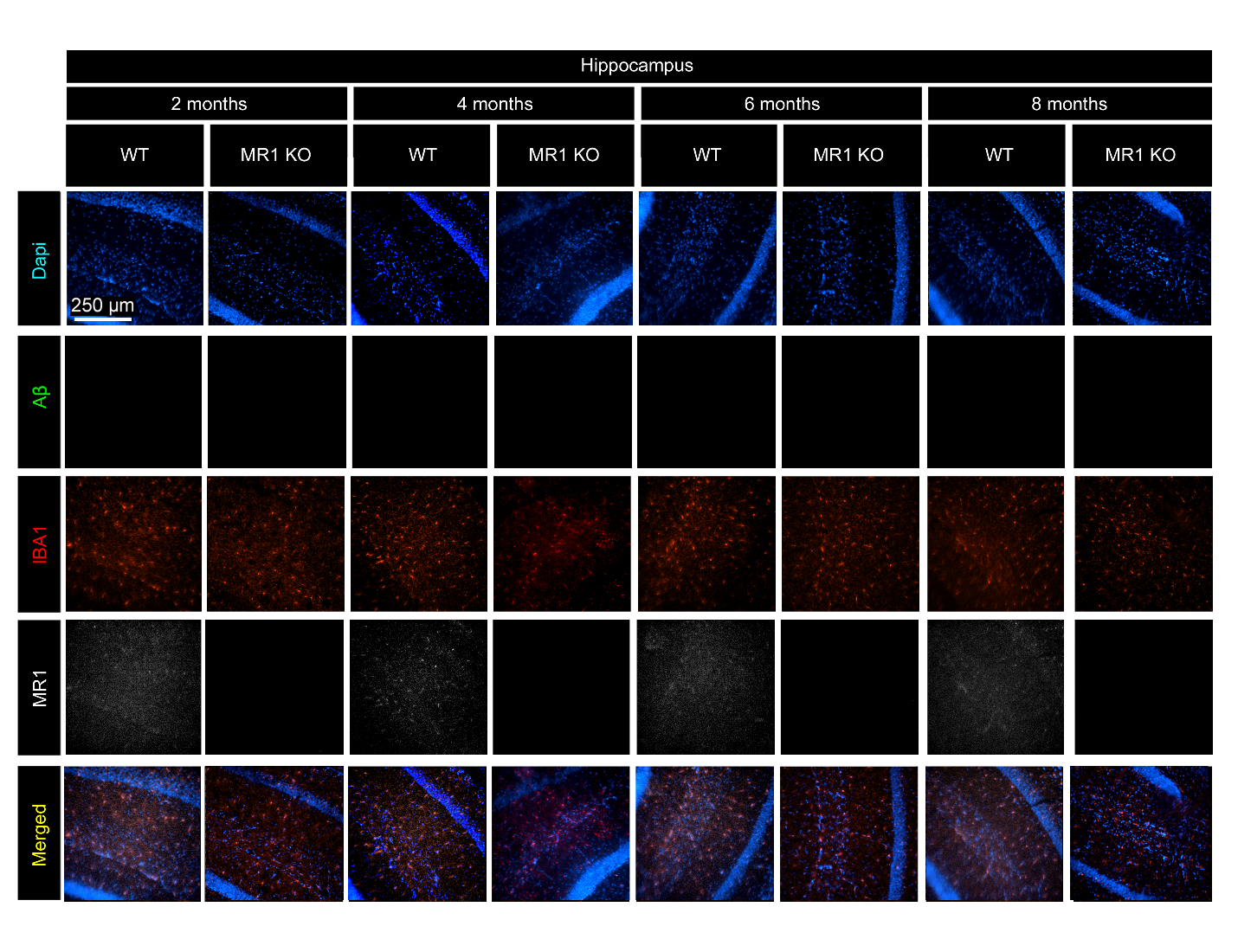
**

**Figure S3.** Immunofluorescent microscopy of the hippocampal CA1 region from wild-type (WT) and MR1 KO mice used for the densitometry analysis shown in Figure 3. Representative images show Dapi (blue), IBA1-labeled microglia/macrophages (red), Aβ (green), MR1 (white), and the images merged (**B**). Scale bar = 250 µm.

**Figure S4**


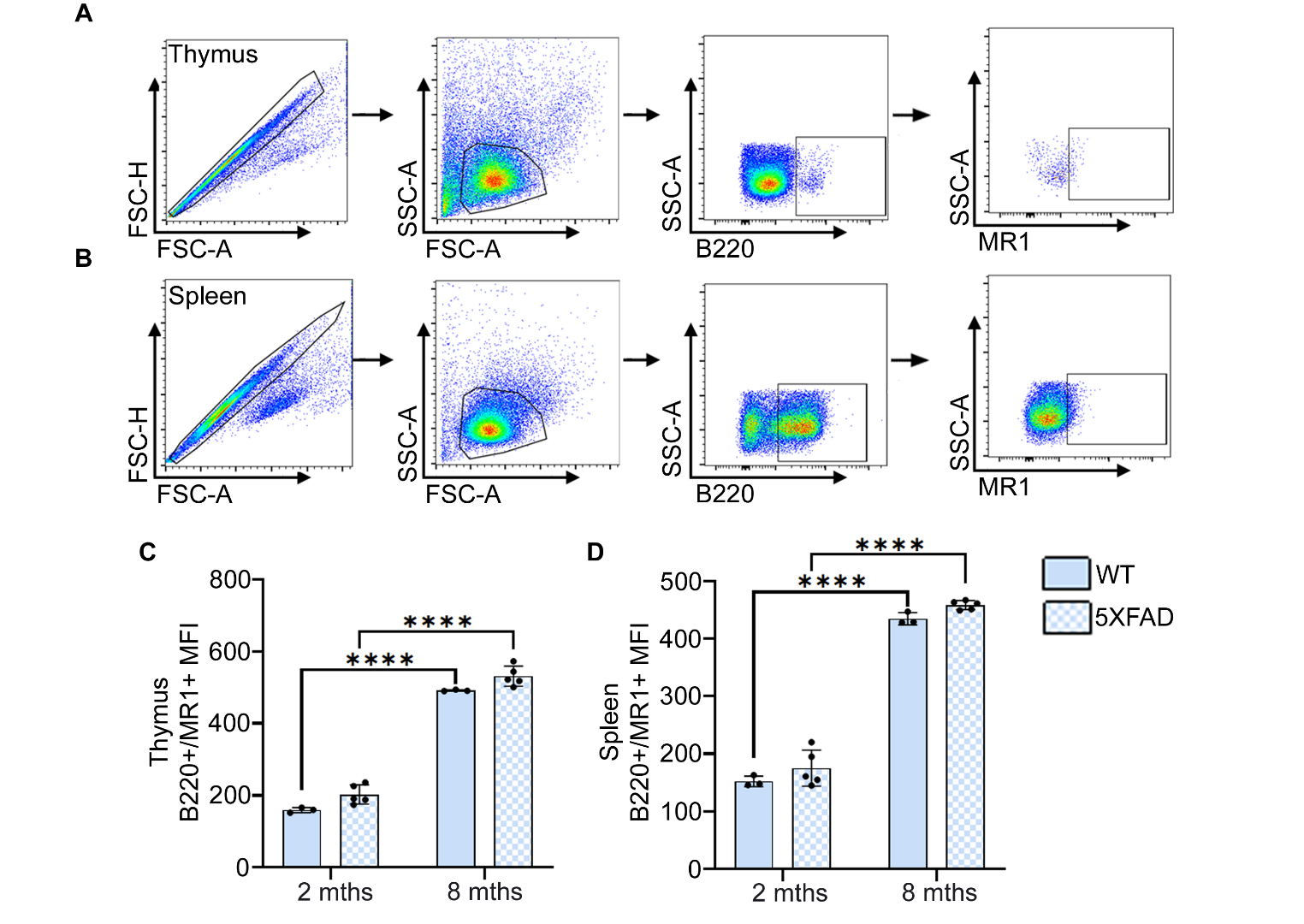


**Figure S4.** MR1 expression on B cells in the thymus and spleen. **A-B** Representative flow cytometry panels showing the gating strategy for the identification of MR1^+^ B cells in the thymus (**A**) and spleen (**B**). **C-D** Mean fluorescent intensity (MFI) of MR1 on B cells in the thymus (**C**) and spleen (**D**). Statistical analysis was performed using a two-way ANOVA with Tukey post hoc multiple comparison test. *****P* < 0.0001 (*n* = 3-4/group). The data are shown as the mean ± standard error of the mean.

**Figure S5**


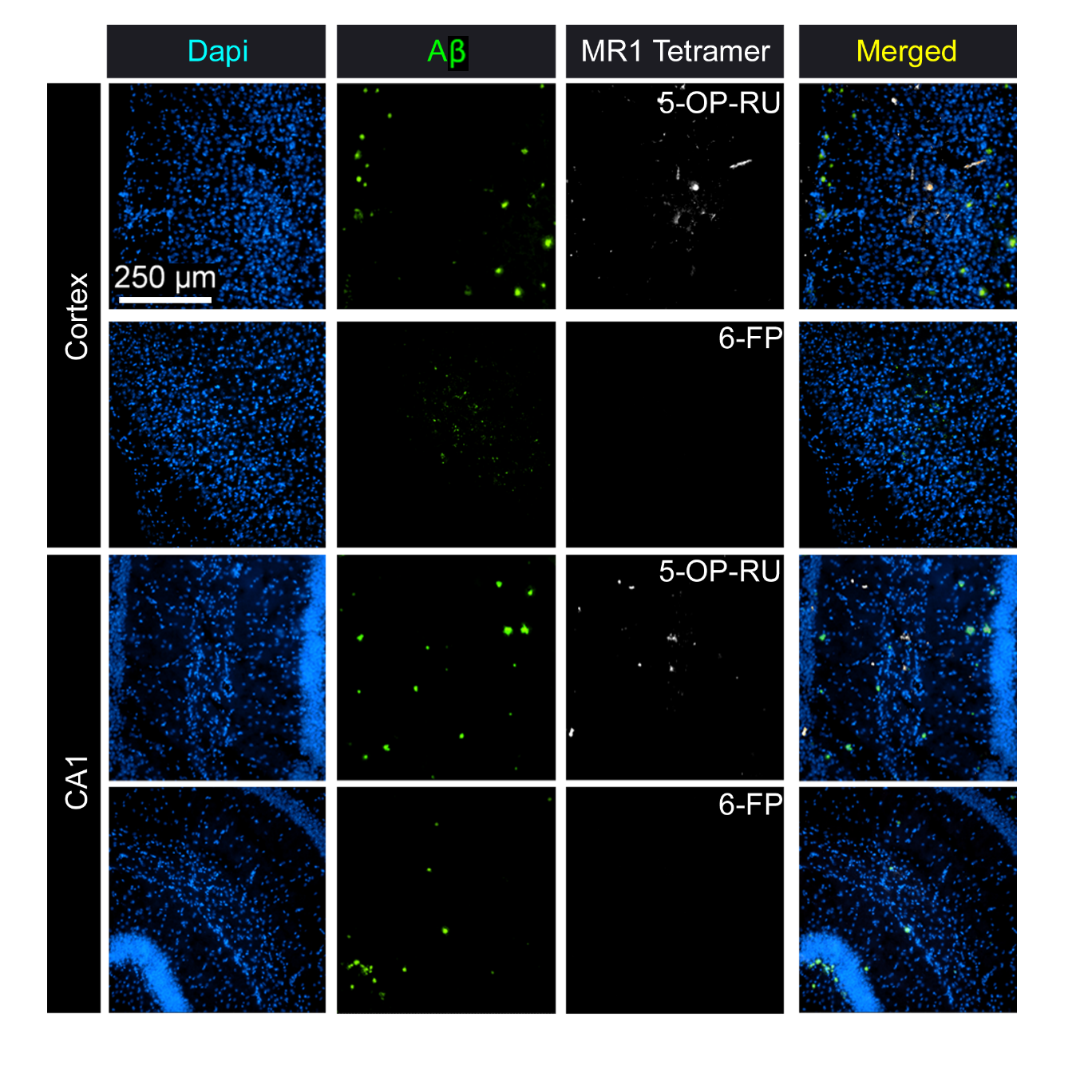


**Figure S5.** Specificity of MAIT cell staining using MR1 tetramers. Representative images of 5XFAD mouse brain sections stained with 5-OP-RU-loaded and 6-FP-loaded (negative control) MR1 tetramers. The images show Dapi (blue), Aβ (green), 5-OP-RU or 6-FP-loaded MR1 tetramers (white), and the images merged. Scale bar = 250 µm.

**Figure S6**


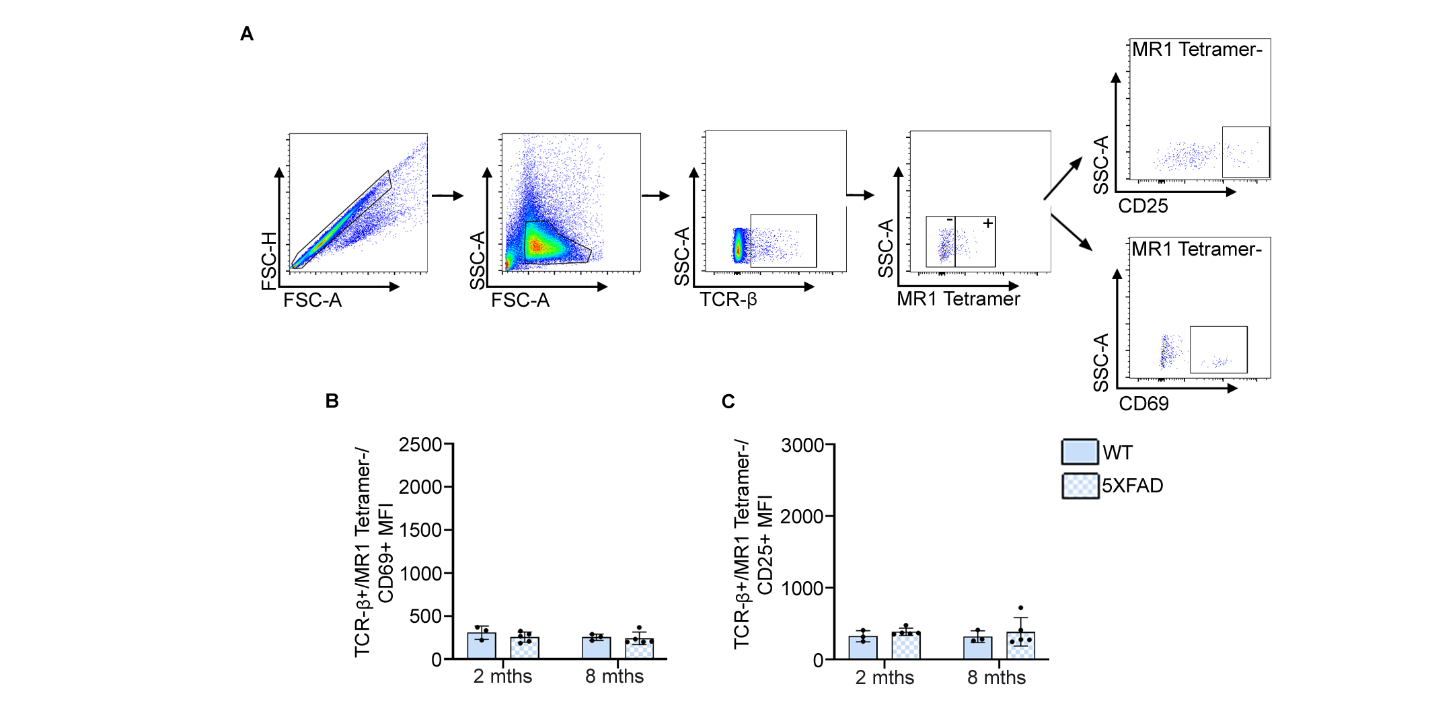


**Figure S6.** Non-MAIT T cells in the brain express substantially lower levels of CD69 and CD25 (compared to Fig. 5F, G). **A** Representative flow cytometry panels showing the gating strategy for identification of TCRβ^+^ T cells that are MR1 tetramer- and their activation state (CD69^+^ or CD25^+^). **B-C** Mean fluorescent intensity (MFI) of CD69 (**B**) and CD25 (**C**) expression on non-MAIT T cells. Statistical analysis was performed using a two-way ANOVA with Tukey post hoc multiple comparison test. (*n* = 3-4/group). The data are shown as the mean ± standard error of the mean.
